# Predicting return of lung function after a pulmonary exacerbation using the cystic fibrosis respiratory symptom diary-chronic respiratory infection symptom scale

**DOI:** 10.1186/s12890-024-03148-w

**Published:** 2024-07-24

**Authors:** Eliana R. Gill, Christopher H. Goss, Scott D. Sagel, Michelle L. Wright, Sharon D. Horner, Julie A. Zuñiga

**Affiliations:** 1 Division of Biobehavioral Nursing and Health Informatics, Department of Nursing, University of Washington, Seattle, WA USA; 2 Division of Pulmonary, Critical Care, and Sleep Medicine, Department of Medicine, University of Washington, Seattle, WA USA; 3 Division of Pulmonary and Sleep Medicine, Department of Pediatrics, University of Washington, Seattle, WA USA; 4 Seattle Children’s Research Institute, Seattle, WA USA; 5 Department of Pediatrics, Children’s Hospital Colorado, University of Colorado School of Medicine, Aurora, CO USA; 6 The University of Texas at Austin School of Nursing, Austin, TX USA

## Abstract

**Background:**

Pulmonary exacerbations (PExs) in people with cystic fibrosis (PwCF) are associated with increased healthcare costs, decreased quality of life and the risk for permanent decline in lung function. Symptom burden, the continuous physiological and emotional symptoms on an individual related to their disease, may be a useful tool for monitoring PwCF during a PEx, and identifying individuals at high risk for permanent decline in lung function. The purpose of this study was to investigate if the degree of symptom burden severity, measured by the Cystic Fibrosis Respiratory Symptom Diary (CFRSD)- Chronic Respiratory Infection Symptom Scale (CRISS), at the onset of a PEx can predict failure to return to baseline lung function by the end of treatment.

**Methods:**

A secondary analysis of a longitudinal, observational study (*N* = 56) was conducted. Data was collected at four time points: year-prior-to-enrollment annual appointment, termed “baseline”, day 1 of PEx diagnosis, termed “Visit 1”, day 10–21 of PEx diagnosis, termed “Visit 2” and two-weeks post-hospitalization, termed “Visit 3”. A linear regression model was performed to analyze the research question.

**Results:**

A regression model predicted that recovery of lung function decreased by 0.2 points for every increase in CRISS points, indicating that participants with a CRISS score greater than 48.3 were at 14% greater risk of not recovering to baseline lung function by Visit 2, than people with lower scores.

**Conclusion:**

Monitoring CRISS scores in PwCF is an efficient, reliable, non-invasive way to determine a person’s status at the beginning of a PEx. The results presented in this paper support the usefulness of studying symptoms in the context of PEx in PwCF.

## Introduction

Cystic fibrosis (CF) is one of the most common, lethal genetic disorders in the United States, and affects almost 40,000 people [1]. The major health risk for people with CF (PwCF) is acute pulmonary exacerbation (PExs), which is characterized by worsening respiratory symptoms, and associated with decreased quality of life, morbidity and mortality [2–4]. PExs are also a major driver of healthcare costs, with the cost of a single episode ranging from $60,800-$74,830 [5]. PExs increase the risk for decline in lung function: it has been shown that 25% of people who are treated for a PEx fail to recover to their baseline lung function by 3 months after antibiotic treatment [6]. Further, PExs are associated with 50% of permanent decline in lung function, and the more frequently PwCF experience PExs the more rapid the decline and risk for respiratory failure [7–9]. Therefore, it is critical to understand what characteristics can predict failure to recover baseline lung function.

When PwCF are diagnosed with a PEx they experience an increase in physiological symptoms or symptom burden [10, 11]. Symptom burden results from the continuous physiological and emotional symptoms related to the disease and/or treatment for a PEx [12]. Higher symptom burden in chronic illnesses is associated with an increase in healthcare utilization [13, 14]. In chronic obstructive pulmonary disease, people with high symptom burden had significantly increased healthcare utilization and were more likely to have died by the 5-year follow-up than people with moderate or low symptom burden [15].

Symptom burden is often measured in PwCF to evaluate the efficacy of treatment when PExs occur. Symptom burden, as measured by the Cystic Fibrosis Respiratory Symptom Diary (CFRSD)-Chronic Respiratory Infection Scale (CRISS) in PwCF has been associated with c-reactive protein (CRP), a marker of systemic inflammation [16], suggesting that symptom burden increases in response to inflammation or infection, and may be a useful measure for predicting treatment outcomes. The change in symptom burden CRISS score from a person’s baseline during a PEx is a viable efficacy endpoint in clinical trials [17]. However, symptom burden score at the onset of PEx therapy has not been analyzed as a predictor of lung function loss in PwCF and may help us to identify people at increased risk for poor recovery from PExs.

We hypothesize that a higher symptom burden, as measured by the CFRSD-CRISS, at the beginning of a PEx may predict people who will not recover their lung function by the end of treatment, and identify a group of individuals at risk for permanent decline in lung function. Therefore, the purpose of this study was to investigate if the degree of CRISS score severity at the onset of a PEx can predict failure to return to baseline lung function by the end of treatment.

## Methods

### Study population

This study is a secondary analysis of a longitudinal, observational study that explored changes in clinical outcomes and systemic measurements of inflammation in response to antibiotic therapy for PExs in PwCF [18]. The original study enrolled 123 participants from 6 CF Foundation (CFF) Centers in the United States from 2007 to 2009 who were diagnosed with an acute PEx; defined as presenting at least 3 of 11 criteria for PEx and requiring at least 2 IV antibiotics. Members of the original study attempted to collect data prior to the administration of IV antibiotics, but initiation of antibiotics was not an exclusion criterion. Data access were acquired from the CFF Therapeutics Development Network Coordinating Center (TDNCC), in Seattle, Washington after review and approval. Data from the original study and matched CFF registry data were provided. Inclusion criteria for the study included PwCF 10 years of age and older. We further restricted our analysis to those with symptom data gathered on Day 1 of PEx diagnosis, and percent-predicted forced expiratory volume in 1 s (ppFEV_1_) data gathered at the previous year’s annual visit and as well as Day 10–21 of PEx diagnosis. No data received from the TDNCC contained personally identifiable information or qualifying HIPAA identifiers, and thus The University of Texas at Austin’s Institutional Review Board (IRB) determined that this study met the criteria for exemption from IRB review under 45 CFR 46.104 (4) secondary research data or specimens (no consent required) (IRB ID: STUDY00000967).

### Timing of assessments and measures

For the present study we used data from four time points: (1) annual well-visit data from the year prior to enrollment into the study, termed annual visit; (2) the first 24-hours of IV treatment for PEx, termed Visit 1; (3) end or near-end of treatment, typically between day 10–21 of IV antibiotic treatment for PEx, termed Visit 2; (4) two-weeks post-hospitalization and cessation of IV and oral antibiotic treatment, termed Visit 3. We measured seven demographic variables: age, sex, race, ethnicity, health insurance, smoking history and exposure to secondhand smoke; and six CF-related variables: ppFEV_1_, homozygous delF508 mutation, positive *Pseudomonas aeruginosa* infection as reported on the annual visit, nutritional status, nights spent in the hospital, and nights spent on home IV antibiotics.

Symptoms and burden were collected and measured using the CFRSD-CRISS, which is composed of eight items. The response for each item is scored on a 5-point Likert-scale, ranging from 0 for no symptoms to 4 for extremely severe symptoms, which address: difficulty breathing, feeling feverish, having chills/sweats, increased cough, increased mucus production, fatigue, chest tightness, and wheezing [19]. Participants missing more than 1 response to the CFRSD were removed from analysis for incomplete data. Each item response has a corresponding score that is totaled for a raw summated score, ranging from 0 to 24. The raw summated score is then converted to a continuous Rasch-logit score, ranging from 0 to 100, termed the CRISS score. The higher the CRISS score the worse the symptom burden.

Spirometry measures were gathered at the annual visit, Visit 1 and Visit 2, and the ppFEV_1_ was calculated using reference Eqs. [20, 21]. Failure to recover baseline lung function by Visit 2 was calculated as the ppFEV_1_ at Visit 2 minus the ppFEV_1_ at the annual visit. A result of 0 indicated a return to baseline lung function, a positive result indicated improved recovery of ppFEV_1_ and a negative result indicated failure to recover baseline ppFEV_1_ by Visit 2.

### Statistical analysis

Data were assessed for normality and missingness. Descriptive statistics were used to examine the mean and standard deviation of continuous variables and frequencies for categorical variables. We used linear regression to test the relationship between CRISS score and lung function recovery. We controlled for age, gender, and *P. aeruginosa* infection due to research showing that PwCF who are female, older and have chronic *P. aeruginosa* infection are more likely to respond to a lesser extent to PEx treatment [6, 22]. We visualized the distribution of residuals; measured the covariance ratio levels for levels outside 1 plus three times the leverage and the residuals for values greater than 3; and tested for independence of variable, outliers, the homogeneity of variance and multicollinearity. R version 4.2.1 was used for all statistical analysis.

## Results

A total of 56 participants were included in this analysis; attrition was related to missing ppFEV_1_ data (*n* = 67). The demographic and clinical characteristics of the included cohort can be found in Table 1 and were compared to participants not included. This subset was representative of the overall study cohort. The majority of participants were Non-Hispanic White (*n* = 52, 92.9%), female (*n* = 37, 66%) and the mean age was 25.1 (*Standard Deviation [SD]* = 9.81) years. The majority of participants were positive for *P. aeruginosa* (*n* = 44, 78.6%). The median length of hospitalization was 16 nights, and the median time spent on home IV antibiotics was 12 nights. The majority of our participants did not use oxygen throughout the year (*n* = 38, 67.9%) or at the beginning of PEx treatment (*n* = 49, 91.5%), and no participants smoked (*n* = 0, 0%). We assessed body mass index (BMI) as a measure of nutritional status for participants 21 years of age and older, and the Center for Disease Control clinical growth charts weight-for-age for participants 12–20. The mean BMI for participants 21 years of age and older was 20.71 kg/m^2^ (*SD* = 2.63), and the majority of participants less than 21 years of age were normal weight-for-age percentile (*n* = 13, 61.9%).

**Table 1:** Demographic characteristics

	Secondary analysis (*N* = 56)			Parent Study (*N* = 123)		
Demographic Variables	*N* (%)			*N* (%)		
Age						
10–20	21 (37.5%)			61 (50.8%)		
20+	35 (62.5%)			59 (49.2%)		
Gender, Female	37 (66.7%)			72 (60%)		
Race/Ethnicity						
White	52 (92.95%)			116 (94.3%)		
Non-Hispanic	51 (91.1%)			113 (91.8%)		
Hispanic	1 (1.8%)			3 (2.4%)		
Black	2 (3.6%)			2 (1.6%)		
Bi-Racial	2 (3.6%)			2 (1.6%)		
Insurance Type						
Medicaid	31 (53.6%)			43 (35%)		
Medicare	5 (8.9%)			13 (10.6%)		
Private	21 (37.5%)			54 (43.9%)		
Private & Medicaid	8 (14.3%)			16 (13%)		
Other	3			7 (5.7%)		
No Insurance	0			1 (0.8%)		
Smokes	0 (0%)			0 (0%)		
Second-hand smoke	-			-		
Daily	2 (3.6%)			5 (4.1%)		
Several Time per Week	2 (3.6%)			6 (4.9%)		
Several Times per Month	4 (7.14%)			11 (8.9%)		
Never	19 (33.9%)			32 ( 26%)		
Oxygen Use	-			-		
Throughout the Year	18 (32.1%)			12 (10%)		
During of PEx	7 (8.5%)			12 (10%)		
BMI, < 21 years old						
Underweight (< 5%)	6 (28.6%)			7 (18.4%)		
Normal weight (5-85%)	13 (61.9%)			29 (76.3%)		
Overweight (85-99%)	2 (9.5%)			2 (5.3%)		
Homozygous DelF508	35 (62.5%)			66 (53.7%)		
*Pseudomonas aeruginosa* + culture	44 (78.6%)			55 (44.7%)		
	**Mean (SD)**	**Median**	**Min & Max**	**Mean (SD)**	**Median**	**Min & Max**
Age, all	25.3 (9.81)	23	12, 50	22.38 (9.83)	21	10, 58
10–20	16.1 (2.5)	16	12, 20	15.06 (3.33)	16	10, 20
20+	30.8 (8.3)	29	21, 50	29.9 (8.51)	27	21, 58
BMI, 21 + years of age	20.1 (2.6)	22	15.5, 23.8	21.2	20.9	16.1, 33.5
ppFEV_1_	52.3 (20.2)	55.4	17.5, 93.5	54.7 (21.3)	51.9	17.5, 95.5
Hospital Nights	23.9 (23.1)	15.5	0, 87	25 (29.2)	15	0, 194
Adult	22.3 (23.6)	16	0, 87	26.8 (35.8)	13.5	0, 194
Child	26.8 (22.6)	15	3, 72	23.1 (20.2)	17.5	0, 86
Home Intravenous Antibiotics	18.6 (24.8)	11.5	0, 110	16 (25.9)	6	0, 180
Adult	23.6 (27.3)	19	0, 110	22.4 (30.6)	14	0, 180
Child	10.1 (17.5)	0	0, 71	9.2 (17.4)	0	0, 90

**Fig. 1:**
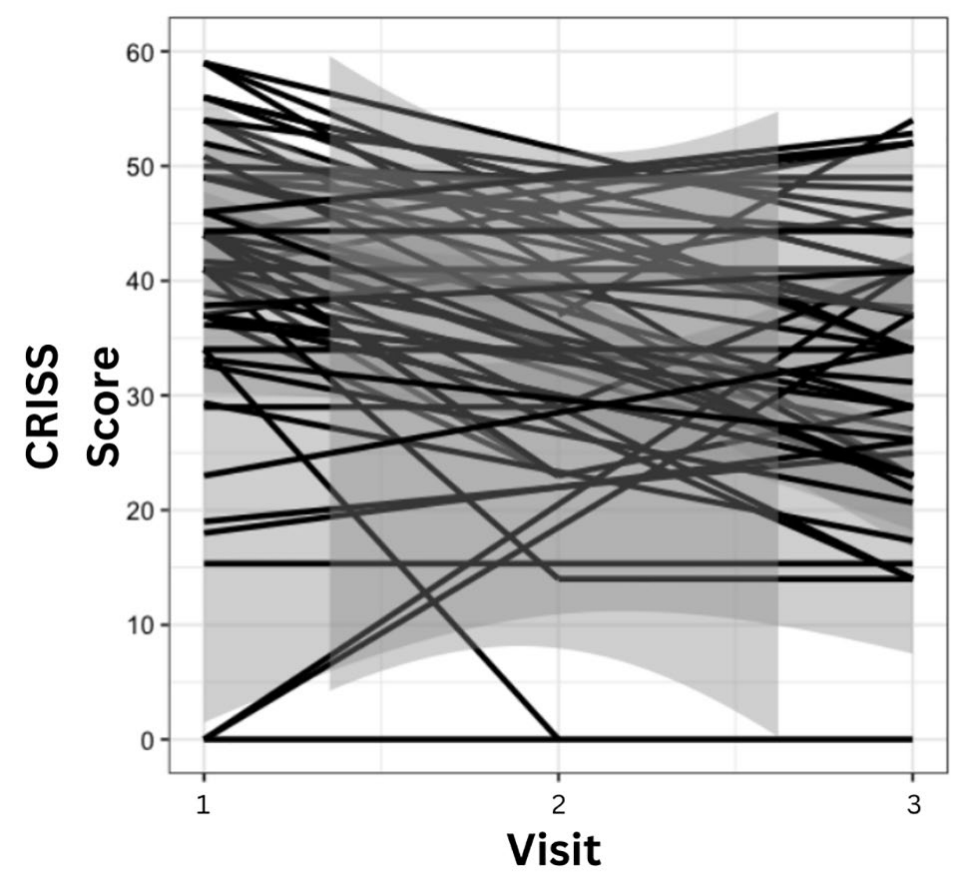
Participant Chronic Respiratory Infection Symptom Scale (CRISS) score over 3 time points during treatment for a pulmonary exacerbation. Visits: 1 = Visit 1, 2 = Visit 2, 3 = Visit 3

The mean ppFEV_1_ at the annual visit was 58.4% (*SD* = 21.6), Visit 1 was 52.28% (*SD* = 20.24) and Visit 2 was 60.59% (*SD* = 23.65). The majority of patients recovered to baseline lung function (*n* = 34, 60.7%), and the average amount of lung function recovered by Visit 2 from the annual visit was 1.52% (*SD* = 4.35, range: -6.28-12.8). However, 39.3% of participants still failed to recover to baseline lung function, and 10.4% failed to recover within 10% of their baseline lung function by Visit 2. The most prevalent symptom experienced by participants was increased cough (*n* = 55, 98.2%), followed by increased mucus (*n* = 49, 87.5%) and fatigue (*n* = 45, 80.4%). The most severe symptom experienced was also increased cough, 2.29 (*SD* = 0.76), followed by fatigue, 2.09 (*SD =* 1.03) then increased mucus, 1.95 (*SD* = 1.05) based on a raw score. A summary of the frequency and severity of measured symptoms can be found in Table 2. The mean CRISS score at Visit 1 was 44.75 (*SD* = 10.67) and at Visit 2 was 23.69 (*SD* = 14.83). Symptom burden significantly improved with IV antibiotic treatment (*p* < 0.001), and while CRISS scores were still significantly better (*p* < 0.01) at the follow-up appointment 2 weeks post-hospitalization, scores increased again after systemic antibiotic treatment was completed (Fig. 1).

**Table 2:** Symptom Prevalence, visit 1

*N* = 56	Frequency (%)	Mean Severity (SD)	Min, Max
Difficulty Breathing	32 (57.1%)	1.23 (1.27)	0, 4
Feverish	11 (19.6%)	0.39 (0.82)	0, 3
Chills/Sweats	10 (17.9%)	0.34 (0.82)	0, 4
Fatigue	45 (80.4%)	2.09 (1.3)	0, 4
Chest Tightness	23 (41.1%)	0.93 (1.26)	0, 4
Cough	55 (98.2%)	2.29 (0.76)	0, 4
Mucus production	49 (87.5%)	1.95 (1.05)	0, 4
Wheezing	24 (42.9%)	0.64 (0.84)	0, 3
CRISS Score	–	44.75 (10.67)	0, 61

### CRISS score and lung function

We found that CRISS score at Visit 1 significantly predicts failure to recover ppFEV_1_ at Visit 2, even when controlling for age, sex, and *P. aeruginosa* infection (Table 3). The model was statistically significant, Adjusted R^2^: 0.14 (*p* < 0.02). The coefficients were intercept = 9.66, CRISS score = -0.2, age = 0.12, *P. aeruginosa* = -1.97, and gender = -1.1. While the model was overall statistically significant, the CRISS score was the only significant predictor within the model (*p* < 0.01) while the three variables we controlled for were not significant (*p* > 0.05). The model results predict that recovery of lung function decreased by 0.2%-predicted points for every increase in CRISS points, indicating that participants with a CRISS score greater than 48.3 were at 14% greater risk of not recovering to baseline lung function by Visit 2, than people with lower scores, when all else is held constant. A post-hoc sensitivity analysis was conducted and included baseline lung function as a control variable; the sensitivity analysis showed that higher CRISS scores continued to significantly predict (*p* < 0.01) failure to return to baseline lung function by Visit 2. Similar to our original analysis, no control variables significantly contributed to the model (*p* > 0.05), yet the overall model continued to be significant, Adjusted R^2^ = 0.14 (*p* < 0.05).

**Table 3:** Linear Regression Model for Return to Baseline ppFEV_1_ outcome

Fixed Effects	Coeff	SE	t	df	95% CI
Intercept (Mean)	9.66	3	3	48	3.6, 14.6**
CRISS Score	-0.2	0.07	-2.8	48	-0.3, -0.1**
*P. aeruginosa*	-1.97	1.2	-1.3	48	-5.2, 1
Age	0.12	0.07	1.8	48	-0.02, 0.2
Gender	-1.1	1.2	-0.9	48	-3.9, 1.1
*Model Fit*					
*R* ^2^	0.14				

Linear regression model output analyzing if Chronic Respiratory Symptom Scale (CRISS) score can predict the return of percent-predicted forced expiratory volume in 1 s (ppFEV_1_)

Note: *N* = 56; Variables were effect coded: *P. aeruginosa* + = 1, *P. aeruginosa* - =0; Male = 1, Female = 0. Model estimates with full information maximum likelihood using R lme4 and lmerTest packages

*p < 0.05, **p < 0.01

## Discussion

In this study of 56 people living with CF, we found that CRISS scores at the onset of PEx significantly predicted failure to recover ppFEV_1_ by the end of systemic antibiotic treatment, even when controlling for age, sex, and *P. aeruginosa* infection. Interestingly, these confounding variables chosen a priori were not significant. It is possible that while these variables have a significant relationship to incomplete PEx treatment response [6] they do not influence the relationship between symptom burden and failure to recover baseline ppFEV_1_ between Days 10–21. Future research should investigate other variables that may influence this relationship such as the use of highly effective CFTR modulator therapy and nutrition status.

The most common symptoms were increased cough, mucus and fatigue; increased cough and fatigue were the two most severe symptoms experienced, and are similar to previous research [23]. Our results support previous findings that, while CRISS scores are sensitive to IV antibiotics, the majority of CRISS scores increased again after treatment was stopped [24]. As VanDevanter et al. (2017) implored, PEx research should not only focus on the improvement in clinical end-points seen at the cessation of antibiotic therapy, but they must prioritize the optimization of longitudinal response to PEx treatment, such as symptom burden and ppFEV_1_. The present study supports that symptoms must be monitored even after the cessation of antibiotic therapy since symptom burden increases again after treatment and a higher overall symptom burden may predict a lower likelihood of returning to baseline after treatment.

Previously, studies have focused on the change in CRISS score during PEx treatment to be used as a reference for end-to-clinical treatment, but none have used symptom burden at PEx onset to predict PwCF at high risk for incomplete treatment response. Our results suggest that the higher the symptom burden at the onset of treatment for a PEx, the lower the chances of returning to baseline ppFEV_1_ at the completion of antibiotic therapy. Based on this model, a participant with a CRISS score at Visit 1 of 48.3 or greater is less likely to regain baseline lung function by Visit 2. The mean CRISS score of these participants is 44.8 and our model suggests that less than one standard deviation (10.8) above the mean has a 14% higher chance of not recovering their baseline lung function by Day 10–21 of their PEx treatment. Three recent studies (combined *N* = 1,146) measured the CRISS score at the onset of a PEx in PwCF, and their mean or median CRISS scores ranged from 49 to 56.6 [16, 17, 25]. This conveys that, on average, PwCF at the beginning of a PEx treated with IV antibiotics consistently have CRISS scores above 48.3. Our results suggest that measuring symptom burden at PEx onset may be a useful tool for identifying PwCF at risk for incomplete treatment response before the end of antibiotic therapy.

There is a need for improved monitoring of lung function following PEx treatment. Heltshe et al. (2023) found that 49.6% of participants who received antibiotic treatment returned to baseline lung function when compared to participants not undergoing PEx therapy [26]. The most improvement in lung function following IV antibiotic therapy has been shown to occur within the first 7–10 days of treatment [27], and any recovered lung function may begin to decline again anywhere between 1 and 16 weeks after therapy has ended [28, 29]. However, as Heltshe et al. (2023) have identified, previous research on recovery of lung function has been obscured by the inherent progression of the CF disease, natural variability in ppFEV_1_ and inconsistent observation times (p. 2), which may have mischaracterized that PExs treatment are inadequate [26]. A weakness of the presented secondary analysis is the lack of long-term follow-up after treatment has ended and no inclusion of comparators. However, PwCF who do not recover their baseline ppFEV_1_ by Day 10–21 of a PEx, a period when they have been receiving IV antibiotics, may be at even higher risk of not recovering to their baseline lung function by 3 months. Utilizing CRISS score we have still identified a group at high risk for failure to recover baseline lung function. Our results support the inclusion of CRISS score in clinical practice to provide an additional measure of an individual’s response to PEx as well as monitoring CF disease progression, and further research should seek to incorporate consistent measurement times and comparative groups.

CRISS score and CRP have been shown to be significantly associated at the beginning of a PEx [17], and CRP has been shown to significantly predict failure to recover baseline ppFEV_1_ when admission levels are greater than 75 mg/L [30]. The significant relationship between CRP and CRISS score, as well as their abilities to independently predict failure to recover baseline lung function, conveys the clinical relevance of symptom burden as an objective, non-invasive predictor and further research is needed to validate these findings for potential incorporation into clinical practice.

### Limitations

Some limitations should be noted. Due to the nature of a secondary analysis, we were limited by the available data and thus were unable to analyze lung function after treatment for PEx had ended. Further, this study was conducted before the use of highly effective modulator therapy (HEMT). More research is needed to assess if CRISS scores can predict failure to return to baseline lung function in the era of HEMT. However, 10% of PwCF are not eligible for HEMT and globally there are people who are unable to access HEMT; the findings presented in this paper are still very relevant for this group of PwCF and may help to identify those at high risk for poor outcomes.

## Conclusion

Measuring symptom burden via the CRISS score is an efficient, reliable, non-invasive way to determine a patient’s status at the beginning of a PEx. Administering the CFRSD-CRISS at the beginning of a PEx may allow clinicians to identify individuals at high risk for not responding to treatment and permanent decline in lung function. By using a patient’s unique symptom experience clinicians may be able to tailor their treatment to the individual to improve lung function recovery between Day 10–21 of a PEx in PwCF. The results presented in this paper support the usefulness of studying symptoms in the context of PEx in PwCF, and future research is needed to validate these findings.

## Data Availability

Datasets generated and/or analyzed during the current study are available from the Cystic Fibrosis Foundation’s Therapeutics Development Network (https://www.cff.org/researchers/therapeutics-development-network). Data are available at no-cost upon application and peer-review.
